# Non-invasive brain stimulation for suicidal ideation: a systematic review and metanalysis of the current literature

**DOI:** 10.3934/Neuroscience.2025018

**Published:** 2025-07-25

**Authors:** Fiammetta Iannuzzo, Fabrizio Turiaco, Vincenzo Messina, Alessandro Magazzù Minutoli, Maria Catena Silvestri, Maria Rosaria Anna Muscatello, Antonio Bruno

**Affiliations:** Department of Biomedical and Dental Sciences and Morphofunctional Imaging, University of Messina, Via Consolare Valeria 1, Contesse, Messina 98125, Italy

**Keywords:** suicide, suicide ideation, NIBS, TMS, Deep TMS, tDCS

## Abstract

Data suggests that the available therapeutic tools are still insufficient to deal with suicidality. Non-Invasive Brain Stimulation techniques (NIBS) have entered the recognized guidelines for therapies in psychiatry due to the advantages related to safety and tolerability. The purpose of this review and meta-analysis is to assess if and how NIBS techniques are used and could be effective in the treatment of suicidal ideation. The search included the Scopus, Pubmed, and Web of Science databases. The word “suicide” was combined with “NIBS”, “transcranial magnetic stimulation” (TMS), “deep TMS” and “transcranial direct current stimulation”. Nine studies met the inclusion criteria and were included in the review. High frequency repetitive TMS (rTMS) protocols were associated with a significant reduction in suicidal ideation, with individual studies reporting improvements ranging from 20% to over 35% on scales such as the scale for suicide ideation (SSI) and the Beck scale of suicide ideation (BSI) (p < 0.01; p < 0.01; p < 0.001). The meta-analysis showed that active rTMS significantly reduced suicidal ideation compared to sham control conditions (Z = 16.79, p < 0.0001). Heterogeneity was high (I² = 99%, chi-square = 473.22, df = 3, p < 0.0001). High frequency rTMS protocols appeared most effective; deep TMS (dTMS) showed mixed results, and only one study utilized transcranial direct current stimulation (tDCS). Due to limited data, no meta-analysis was conducted on dTMS or tDCS studies. Although preliminary findings suggest a potential for NIBS techniques to reduce suicidal ideation, the current evidence is limited by the small number of high-quality studies and heterogeneity in the protocols and outcomes. Therefore, conclusions regarding clinical efficacy should be considered tentative.

## 1. Introduction

The World Health Organization (WHO) estimates that more than 700,000 individuals die by suicide each year. Suicide is a fatal act that represents the willingness of the subject to die, and ranges from suicidal ideation, namely thoughts about ending one's life or letting yourself die, to a suicide attempt, which is a potentially self-injurious behavior associated with some intent to die, or a suicidal event [1].

In this paper, we use the term suicidality to broadly refer to any suicidal event, including suicidal ideation (thoughts about ending one's life or letting oneself die), suicidal behaviors (potentially self-injurious acts with intent to die), suicide attempts (non-fatal act in which a person deliberately tries to end their own life), and completed suicide. This inclusive operational definition allows us to capture the full spectrum of suicide-related outcomes across studies, regardless of the terminology originally used by the authors.

The WHO has declared that reducing suicide-related mortality is a global imperative, to contrast the traditional taboo that has always surrounded suicidal behaviors [2]. This stigma can prevent individuals from seeking help and treatment and make it difficult for healthcare professionals to ask about and address suicidal thoughts [2]. Various risk factors may be associated with an increased risk of suicidality. Some may be modifiable (i.e., mental illness, physical illness, substance use, insomnia, and a loss of hope), while others may not (i.e., family suicide history, stressful life events, and childhood trauma). According to the literature, psychiatric disorders may be associated with an increased risk of suicide. The most relevant psychiatric disorders related to suicide are depression, substance use, and psychosis, alongside anxiety, personality disorders (i.e., borderline personality disorder), and eating- and trauma-related disorders [3]. The likelihood of suicide increases during times of severe illness and is proportional to the illness's intensity. A previous suicide attempt is the most powerful indicator of future attempts, and, in most cases, suicide is carried out on the first or second attempt [4]. Suicidal ideation, especially when accompanied by the search for means to act, stressful life events, such as bullying or financial difficulties, and physical illnesses with a greater impairment in the quality of life, are other risk factors for suicide. Furthermore, substance abuse issues, including alcohol, prescription medications, and illegal drugs, are linked to a heightened risk of suicide, both in adults and adolescents [5].

An assessment of suicidality can be conducted via clinical interviews or structured screening instruments, while, the treatment of suicide may include pharmacotherapy, behavioral therapy, or sending the patient to an emergency department if there is a serious risk of imminent harm. Improved collaborations between primary and secondary care, along with regular post-discharge check-ups, can enhance patient outcomes [2]. Drug treatments include therapies with lithium, especially in patients with bipolar disorder[6]–[8], and clozapine, especially in schizophrenic patients [6]. Esketamine, an N-methyl-D-aspartate (NMDA) receptor antagonist that affects glutamate transmission, is a new treatment developed as an intranasal treatment for patients with treatment-resistant depression and those at immediate risk of suicide, and has the potential to rapidly relieve symptoms of major depression, including suicidal thoughts [9]. Psychotherapeutic treatments, such as cognitive-behavioral therapy and dialectical-behavioral therapy, could be effective in reducing the risk of suicide, especially in individuals with borderline personality disorder [10],[11].

Data shows that the number of annual suicides has remained almost constant year by year [12], and this data could suggest that the available therapeutic tools are still insufficient to deal with this problem. In the context of mental diseases, non-invasive brain stimulation techniques (NIBS), such as transcranial magnetic stimulation (TMS) and transcranial direct current stimulation (tDCS), have entered the recognized new therapies in psychiatry due to advantages related to safety, tolerability, cost-effectiveness, and compatibility with other possible treatments [13]. Repetitive transcranial magnetic stimulation (rTMS) has been included in several recognized clinical guidelines for the treatment of major depressive disorder (MMD), particularly for cases of treatment resistance. For instance, the American Psychiatric Association (APA) Practice Guideline for the Treatment of Patients with MDD recommends rTMS as an evidence-based intervention [14], as do the Canadian Network for Mood and Anxiety Treatments (CANMAT) guidelines [15] and the National Institute for Health and Care Excellence (NICE) guidelines [16]. In addition, rTMS received U.S. Food and Drug Administration (FDA) approval for the treatment of MDD in 2008 [17], and more recently for the treatment of suicidal ideation in MDD using intermittent theta burst stimulation (iTBS) protocols in 2021 [18]. Transcranial direct current stimulation (tDCS) has also shown promising clinical results, which has led to increased requests for an evidence-based review on its clinical effects [19]. These data suggest that tDCS may be considered a promising adjunctive treatment, especially for patients with treatment-resistant depression, and highlight its favorable safety profile and ease of application.

TMS is used for brain stimulation by applying a rapidly changing magnetic field to the scalp, thus allowing for the modulation of neural activity. It is based on the principle of electromagnetic induction and delivers a strong and short-lived magnetic field that induces a perpendicular electrical current [20]. TMS can either increase or decrease neural activity, depending on the specific stimulation protocol used. rTMS can alter local neural activity for a time, and often extends beyond the stimulation period. It successfully complements existing pharmacological and behavioral treatments for some neurological and psychiatric disorders [21]–[23] due to its long-lasting effects and low risk of side effects (i.e., headache and a nonspecific feeling of discomfort) [22]. Deep TMS (dTMS) is an alternative to conventional TMS, thereby utilizing different H-coils to treat multiple psychiatric and neurological conditions with identifiable brain targets. dTMS offers all the benefits of TMS (i.e., no hospitalization or anesthesia is necessary, and side effects are minimal), as well as the added advantage of stimulating deeper brain targets with a wider distribution of the electric field. On the other hand, compared to conventional TMS coils, which directly stimulate targets located up to approximately 1 cm below the skull surface, dTMS can reach targets located up to around 4 cm below the skull surface, depending on the type of H-coil being utilized [24]. In Europe, dTMS using a H1-coil has been approved to treat a range of mental and behavioral conditions [24], although the role and effect of TMS in the treatment and prevention of suicidality linked to numerous psychiatric diseases is still not clear.

tDCS is a neurostimulation technique based on the passage of a weak current (1–2 mA) across the cortex using at least two electrodes [25].

The effects of tDCS stem from the modification of the conductivity of sodium and calcium' channels and the shift of electrical gradients that affect the ion balance inside and outside the neuronal membrane, thus modulating its activation threshold. A meta-analysis of tDCS for depression suggested that the technique might be effective for depression [26], but further evidence is necessary to explore the effect on suicidality.

NIBS techniques could be used in suicidality treatments thanks to the modulatory activity exerted on the dorsolateral prefrontal cortex (DLPFC), which is a key hub in networks responsible for executive functions, emotion regulation, and inhibitory control [27]–[29]. Indeed, dysfunction in these circuits is strongly implicated in the neurobiology of suicidal ideation and behaviors [30],[31]. NIBS may exert therapeutic effects by normalizing hypoactivity in the prefrontal cortex and improving its connectivity with limbic regions (e.g., the amygdala and the anterior cingulate cortex), thereby enhancing emotional regulation and reducing depressive symptoms [32],[33]. Additionally, NIBS can induce neuroplastic changes, thus promoting synaptic strengthening and network reorganization that prevent maladaptive neural patterns linked to suicide risk [34],[35]. These neurobiological effects provide a plausible mechanism for the observed clinical benefits of NIBS on suicidal ideation.

This work aims to examine the impact of non-invasive neuromodulation treatments on suicidality in neuropsychiatric disease. Given these considerations, the purpose of this review and meta-analysis is to assess if and how NIBS techniques are used and could be effective in the treatment of suicidal ideation.

## 2. Materials and methods

### 2.1. Review protocol

The systematic search was conducted in accordance with the Preferred Reporting Items for Systematic Reviews and Meta-Analyses (PRISMA) guidelines [36]. Our review protocol was pre-registered on January 8, 2025, and registered on January 19, 2025 (PROSPERO registration number: CRD42025635883). No changes were made to the original protocol submitted.

### 2.2. Literature search

A comprehensive literature search was conducted across the Scopus, PubMed, and Web of Science databases. The search terms included the following: “suicide,” “suicidality,” “suicidal ideation,” “suicidal thoughts,” and “suicidal behavior”, which were combined with terms related to neuromodulation, such as “non-invasive brain stimulation,” “NIBS,” “transcranial magnetic stimulation,” “deep TMS,” and “transcranial direct current stimulation”. The search strategy employed a combination of controlled vocabulary (subject headings) and free-text keywords to maximize sensitivity. The full search strings used in each database are reported in Supplementary to ensure transparency and reproducibility.

The publication timeframe for the articles retrieved spanned from the inception of these databases to January 8, 2025, which was the date of the investigation.

### 2.3. Data extraction and synthesis

We conducted a preliminary search, which revealed 290 papers. Articles were included in the review according to the following inclusion criteria: English language, publication in peer reviewed journals, and articles about studies performed on NIBS techniques related with suicidal ideation.

Two independent reviewers extracted data from the included studies using a pre-defined data extraction form. Discrepancies were resolved through a discussion or consultation with a third reviewer. No automation tools were used for data extraction.

#### 2.3.1. Qualitative synthesis

After the duplicates were removed, 246 articles remained. Of these, at the first screening, conducted by title and abstract, 158 studies were excluded. After the second screening conducted by full-text examination, 79 articles were excluded.

Studies were included according to the following PICOS criteria:

- Population (P): Individuals identified as being at risk of suicide, including those presenting with suicidal ideation or behaviors and those diagnosed with any psychiatric condition;

- Intervention (I): Brain stimulation techniques, such as TMS and tDCS;

- Comparison (C): Control conditions including sham stimulation, placebo, or treatment as usual;

- Outcomes (O): Suicidality-related outcomes, including changes in suicidal ideation, suicidal behavior, or overall suicide risk; and

- Study design (S): Only randomized controlled trials (RCTs) were considered eligible for inclusion.

Articles were excluded if they were reviews, meta-analyses, not specific or irrelevant to the topic, whether they involved invasive stimulation techniques such as Electroconvulsive Therapy (ECT), Magnetic Seizure Therapy (MST) and Deep Brain Stimulation (DBS), if they were not RCTs, if they had no sham control group, or if the full text was unavailable.

**Figure 1.**
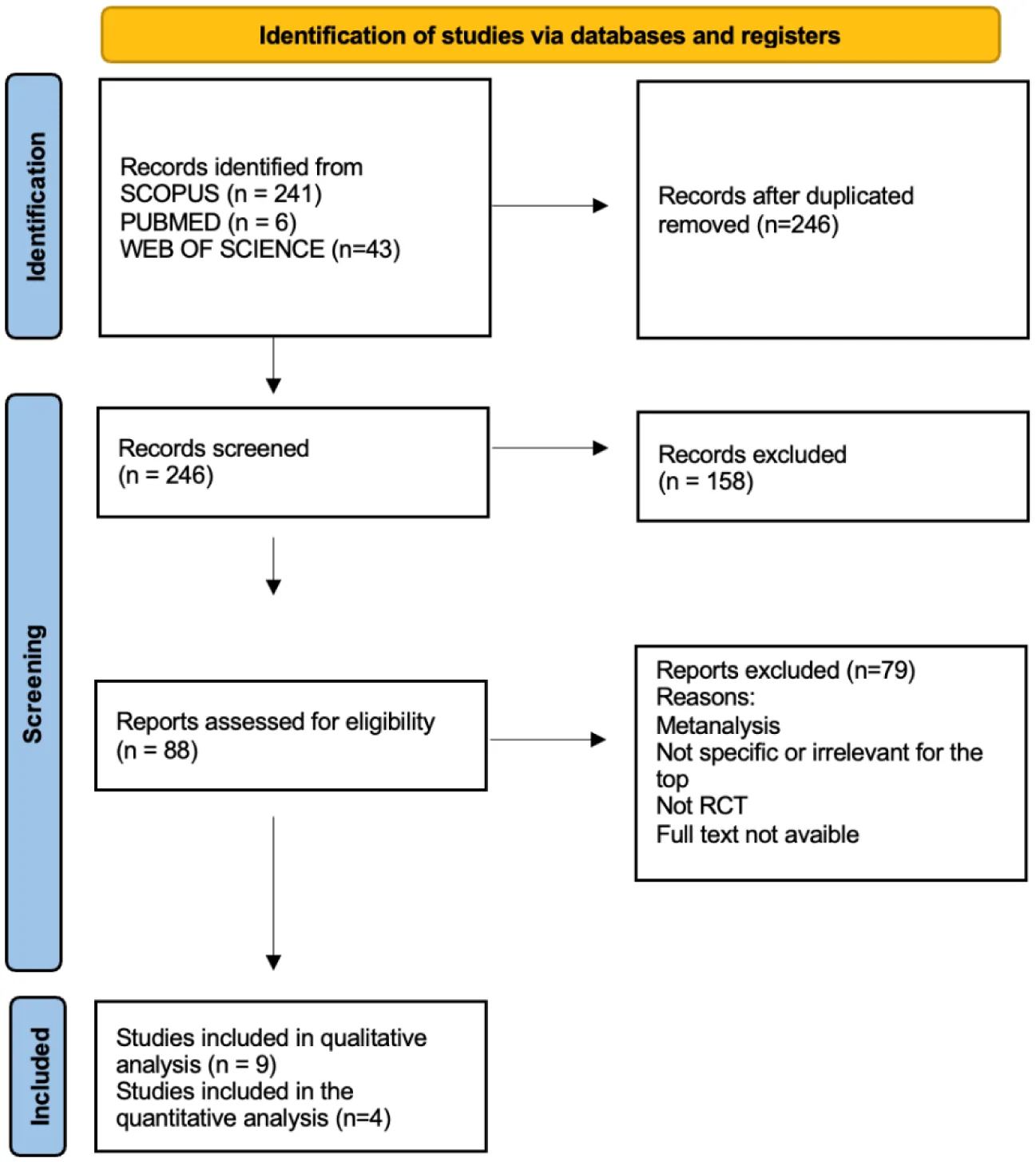
PRISMA 2020 flow diagram for new systematic reviews which included searches of databases and registers only.

Eventually, 9 studies met the inclusion criteria and were included in the qualitative analysis. The annexed table summarizes the selected articles (Table 1), whereas the annexed flow diagram (Figure 1) summarizes the selection process.

We included studies that enrolled individuals at risk of suicide, regardless of specific psychiatric diagnosis, in line with the transdiagnostic nature of suicidality. Indeed, suicidal ideation and behaviors are known to occur across a wide range of mental disorders, including, but not limited to, mood disorders, personality disorders, psychotic disorders, and substance use disorders. However, all the studies that met our inclusion criteria focused on patients with MDD, including treatment-resistant depression in some cases. Regarding severity, the included participants typically presented with moderate to severe depressive symptoms and current suicidal ideation or behaviors. In the only study that employed tDCS, the intervention was used as an add-on to ongoing pharmacological treatment, and not as a monotherapy for severe depression, which is not ethically acceptable at present.

For the outcome assessment, we extracted data at the endpoint of the intervention. The primary analysis considered change-from-baseline scores for the main outcome measures. When multiple time points or analyses were reported, we prioritized the primary outcome and the time point closest to the end of the intervention, as specified in each study.

#### 2.3.2. Quantitative synthesis

In this intervention review, we assessed the efficacy of tDCS and rTMS and compared the control and experimental groups through meta-analyses. We selected only four studies for the meta-analysis due to the homogeneity of the control groups. Studies included in the meta-analysis were selected based on the homogeneity of clinical and control groups. Studies with different types of control groups or heterogeneous clinical populations were excluded from the quantitative synthesis to avoid bias and maintain comparability.

The inclusion criteria included the application of brain stimulation interventions, participants with a risk of suicide, and studies that compared the efficacy of the control groups versus the experimental groups. Meta-analyses were performed using the Review manager package, 5.4.

Effect size calculations were based on the effect of neuromodulation techniques in reducing suicide ideation.

Heterogeneity among the results was explored across the included studies and was assessed using both the Chi-square (χ²) test and the I² statistic.

**Table 1.** Selected studies.

	**Reference**	**Sample size and diagnosis**	**NIBS pattern**	**Control group**	**Psychiatric assessment for suicide**	**Main findings**
rTMS studies and Ai TBS protocols	George et al., 2014 [37]	41 patients with depressive episode (mean age 41.5, S.D. 15.7)	High frequency rTMS protocol on left prefrontal cortex. 9 sessions over 3 days (3 sessions/day, 10 Hz rTMS)	Active sham control group	Scale for suicide ideation (SSI)	SSI scores declined rapidly in both groups with a trend for more rapid decline in the active rTMS (p: 0.12 in the mITT analysis; p: 0.054 in the completers' analysis); however, this difference normalized at days 2 and 3
	Desmyter et al., 2016 [38]	50 patients with treatment resistant depression (mean age 41.90, SD 11.77)	Ai TBS protocol on the left dorsolateral prefrontal cortex. **20 sessions over 4 days** (5 sessions/day, accelerated iTBS, 50 Hz)	Active sham control group	Beck Scale of Suicide ideation (BSI)	Significant decrease of BSI score (p < 0.01) between T1 and T2
	Baeken et al., 2017 [39]	44 patients with treatment resistant depression (mean age 38.73, SD 11.65)	Ai TBS protocol on the left dorsolateral prefrontal cortex **20 sessions over 4 weeks** using 20 Hz dTMS	Passive sham control group	Scale for suicide ideation (SSI)	Decrease in SSI score (p: 0.07)
	Yesavage et al., 2018 [40]	164 patients with treatment resistant depression (mean age 55.2, S.D. 12.4)	High frequency rTMS protocol on the left prefrontal cortex **30 sessions over 6 weeks** (daily 10 Hz rTMS)	Passive sham control group	Scale for suicide ideation (SSI)	Non-significant findings were seen in measures of suicidal ideation at both the end of acute treatment and follow-up phases (p: 0.53)
	Dai et al., 2020 [41]	103 elderly patients with depression and suicidal ideation (mean age 68.2, S.D. 9.37)	High frequency rTMS protocol on the left prefrontal cortex **10 sessions over 2 weeks** (daily 10 Hz rTMS)	Active sham control group	Self-rating Idea of Suicide Scale (SIOSS)	Significant lower SIOSS score in rTMS group compared with sham rTMS group after 2 weeks treatment (p < 0.05) and 4 weeks treatment (p < 0.01)
	Pan et al., 2020 [42]	50 patients with major depressive disorder (mean age 19.79, SD 5.55)	High frequency rTMS protocol on the left dorsolateral prefrontal cortex **15 sessions over 3 weeks** (daily 10 Hz rTMS)	Active sham control group	Beck Scale of Suicide ideation (BSI)	The active rTMS group showed a significantly greater BSI score reduction compared with the sham group at day 7 (p < 0.001)
Deep TMS studies	Berlim et al., 2014 [43]	17 patients with treatment resistant depression (mean age 47.12, S.D. 13.26)	Deep TMS on the left dorsolateral prefrontal cortex **20 sessions over 4 weeks** (20 Hz dTMS)	Passive sham control group	Scale for suicide ideation (SSI)	Reduction of depressive symptoms and suicide ideation after treatment (p: 0.019)
	Kaster et al., 2018 [44]	58 patients with major depressive disorder (mean age 65.21, S.D. 5.5)	Deep TMS on the dorsolateral and ventrolateral prefrontal cortex bilaterally **20 sessions over 4 weeks**, at 18 Hz dTMS	Passive sham control group	Scale for suicide ideation (SSI)	SSI, did not differ significantly between the active and sham rTMS conditions
tDCS studies	Brunoni et al., 2014 [45]	120 patients with an acute major depressive episode (mean age 46.4, S.D. 14). Each patient receved a fixed dose of 50 mg/d of sertraline	tDCS (the anodic stimulation on left and the cathodic stimulation on the right dorsolateral prefrontal cortex) 10 sessions over 2 weeks (2 mA)	Active sham control group	Item 10 MADRS	Patients receiving tDCS and sertraline compared to placebo had significant improvement on MADRS items 1, 2, 6, 7, 8, 9 and 10 (p < 0.01 for all comparisons)

### 2.4. Assessment of risk of bias

To assess the risk of bias in the findings, we used RoB, version 2.0, which is a revised Cochrane risk of bias tool for randomized trials [46]. RoB 2.0 assesses the following domains: randomization process, deviations from intended interventions, missing outcome data, measurement of the outcome, and selection of the reported result. Two reviewers (F.I. and F.T.) independently assessed the risk of bias for each study, with disagreements resolved by a third author (M.S.). No automation tools were used in the risk of bias assessment. Most studies had an overall low risk of bias, while only one study was rated as having a high risk of bias, thus suggesting that most included studies are reliable. For data preparation, we extracted means and standard deviations for continuous outcomes. If statistics were missing, then these were derived from other reported data (e.g., standard errors or confidence intervals).

**Figure 2.**
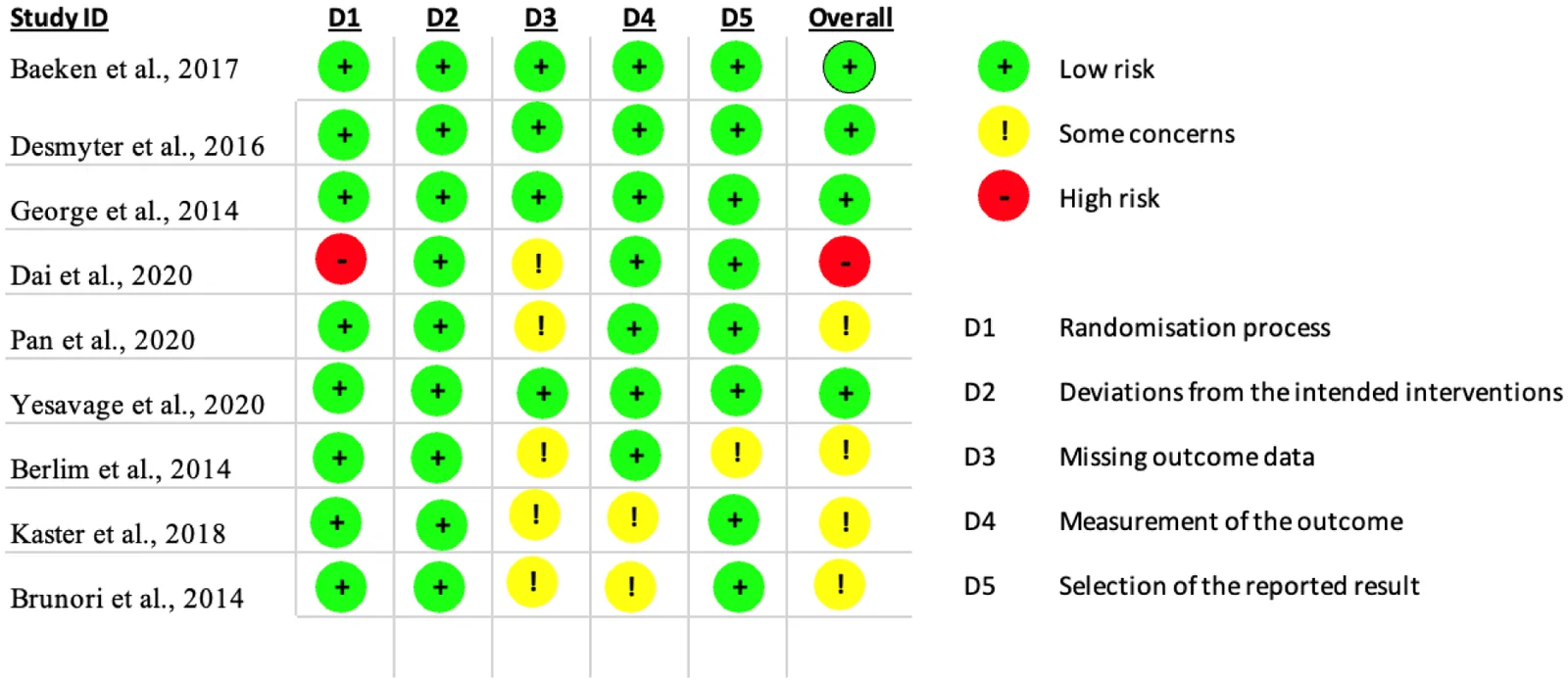
Risk of bias assessment and legend.

Figure 2 shows the risk of bias assessment. Among the nine included studies, five studies [37]–[40],[44] were rated as having an overall low risk of bias, three studies [42],[43],[45] were rated as having some concerns, mainly due to missing outcome data (D3) or concerns related to the selection of the reported result (D5), and one study [41] was judged as having a high overall risk of bias, despite a low risk across most domains, due to issues in the measurement of outcomes (D4). The most frequent source of concern across the studies was domain D3 (missing outcome data), which appeared in four studies. Only one study [37] showed high risk in the randomization process (D1), and one study [41] showed high risk in the outcome measurement (D4).

## 3. Results

### 3.1. Qualitative analysis

#### 3.1.1. Repetitive TMS and ai TBS effect on suicidal ideation

Six studies (sham-controlled clinical trials), 4 studies regarding high frequency rTMS protocols and 2 regarding accelerated intermittent theta burst stimulation (aiTBS) protocols, were included in the present systematic review. In all the high frequency protocol rTMS studies, patients with depressive disorders and suicidal ideation showed an improvement in depressive symptoms and a reduction in suicidal ideation when compared with the sham control group [37]–[42]. The assessment for suicidal ideation was performed through specific instruments: three of them [37],[40],[42] used the scale for suicide ideation (SSI) test, while one of them [41] used the self-rating idea of suicide scale (SIOOS). In all the selected rTMS studies, the left prefrontal cortex was used as the target area in the treatment.

In George et al., [37] the SSI scores rapidly declined in both groups, with a trend for a more rapid decline in the active rTMS (p: 0.12 in the mITT analysis; p: 0.054 in the completers' analysis); however, this difference normalized at days 2 and 3. In Dai et al. [41], significantly lower SIOSS scores in rTMS group compared with the sham rTMS group were detected after 2 weeks of treatment (p < 0.05) and 4 weeks of treatment (p < 0.01). Pan et al., [42] observed how the active rTMS group showed a significantly greater BSI score reduction compared with the sham group at day 7 (p < 0.001). However, in Yesevage et al., [40], non-significant findings were seen in measures of suicidality at both the end of acute treatment and follow-up phases (p: 0.53).

In Baeken et al. [39], patients with treatment-resistant depression underwent accelerated intermittent TBS stimulation on the DLPFC, and showed a change score in “hopelessness” explored by SSI test (p: 0.07). In Desmyter et al. [38], an intensive iTBS protocol was applied over the DLPFC in the treatment of resistant unipolar depressed patients, with a decrease in suicide risk and a significant decrease of the Beck Scale of Suicide ideation (BSI) score (p < 0.01) between T1 and T2.

#### 3.1.2. Deep TMS and effect on suicidal ideation

In 2014, Berlim and his co-workers performed a study in which 17 patients (age: 47.12 ± 13.26) with previously diagnosed treatment-resistant depression (TRD) underwent dTMS [43]. The goal of that study was to determine the effectiveness of dTMS therapy in patients who did not respond well to traditional treatments. The CGI-S scale was used to evaluate the overall symptoms, and the SSI was used to assess suicidality. In this study, the authors underlined a reduction of depressive symptoms and suicide ideation after treatment (p: 0.019). Kaster and colleagues [44] performed a double-blind, randomized, sham-controlled trial in 2018. The participants ranged in age from 60 to 85 years old and had MDD. The study participants were randomly divided into two groups, and either received active or fake rTMS treatment. The active rTMS group was given the following rTMS standardized dose: 18 Hz, at 120% RMT, 2 s pulse train, 20 s interval between trains. The control group received a sham intervention with the same parameters, device, and helmet as the intervention group. Although it has been observed how deep rTMS was associated with a meaningful remission rate, the effect of time on the SSI did not significantly differ between the active and sham rTMS conditions.

#### 3.1.3. tDCS and effect on suicidal ideation

One study [45] described the role of tDCS in suicidality in depressed patients. The results showed that treatment with active tDCS alone was meaningly more effective in decreasing suicidal thoughts than treatment with placebo, and that the combined treatment with active tDCS and sertraline was also better in improving suicidal thoughts than the placebo treatment, all explored on MADRS item 10 (p < 0.01).

### 3.2. Quantitative analysis

For the meta-analysis, we selected only 4 studies with a control group to compare the efficacy of the neuromodulation treatment versus the placebo group. The other studies used for the systematic review were excluded from the meta-analysis because the control and clinical groups were heterogeneous.

Each study included in the meta-analysis contributed to the overall finding that neuromodulation techniques reduce suicidal ideation.

Brunoni et al., [45] reported an effect size of 16.37 (95% CI: 13.35, 19.39), which indicated a statistically significant reduction in suicidal ideation. Dai et al., [41] yielded an effect size of −19.08 (95% CI: −20.62, −17.54); however, its confidence interval overlapped the line of no effect, which indicat4ed no statistically significant impact. Pan et al., 2020 [42] demonstrated a notable effect size of −16.09 (95% CI: −20.66, −11.52), thus showing a significant reduction in suicidal ideation. Yesavage et al., [40] also indicated a reduction, with an effect size of 3.30 (95% CI: −0.78, 7.38), which is consistent with the overall trend.

This variation in individual study results contributes to the observed high heterogeneity (I² = 99%), with a chi-square value of 473.22 (df = 3, p < 0.00001). The differences among studies reflect variability in neuromodulation techniques, participant characteristics, or study methodologies. Overall, these results support the effectiveness of neuromodulation in reducing suicidal ideation, though the high heterogeneity suggests the need for further investigations to understand the sources of variability across studies.

The pooled effect size was statistically significant (Z = 16.79, p < 0.0001), with a 95% confidence interval showing a consistent effect across studies. Heterogeneity was high (I² = 99%, chi-square = 473.22, df = 3, p < 0.0001), thus suggesting substantial variability among studies due to differences in the neuromodulation techniques, study populations, or methodological designs. Overall, these results support the effectiveness of neuromodulation techniques in reducing suicidal ideation, although the high heterogeneity indicates the need for further investigations to clarify the sources of variability across studies.

**Figure 3.**
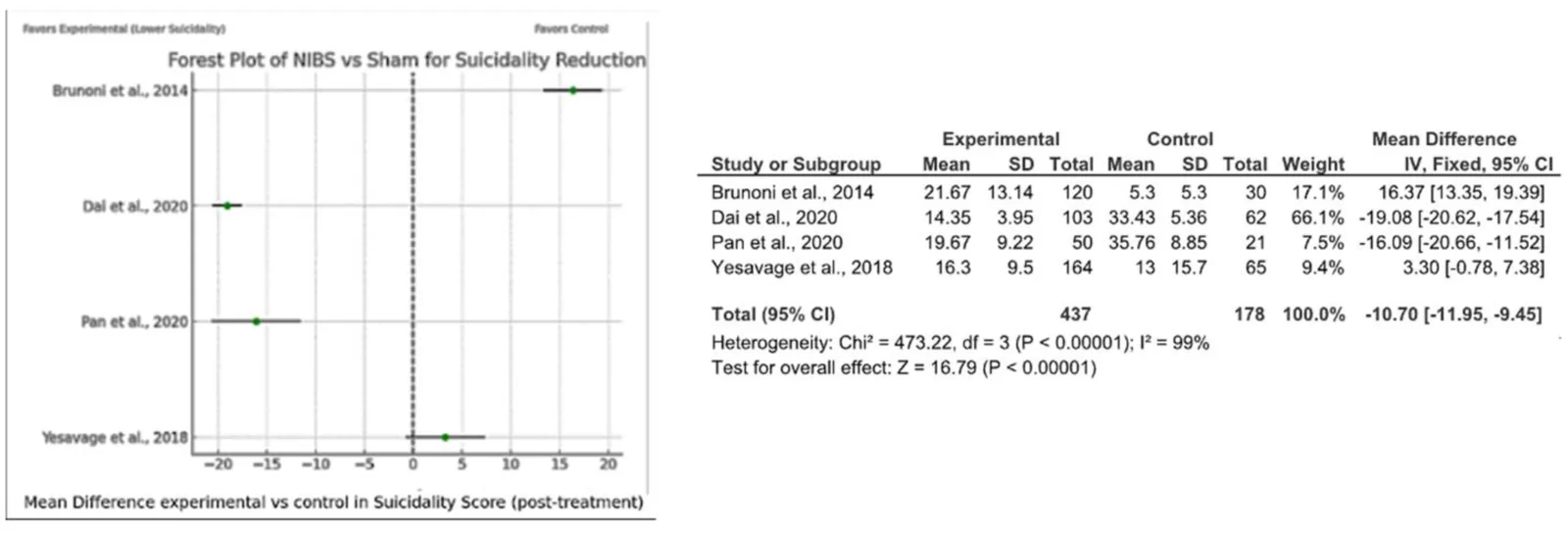
Forest plot showing mean difference from the comparison between two groups and legend.

**Figure 4.**
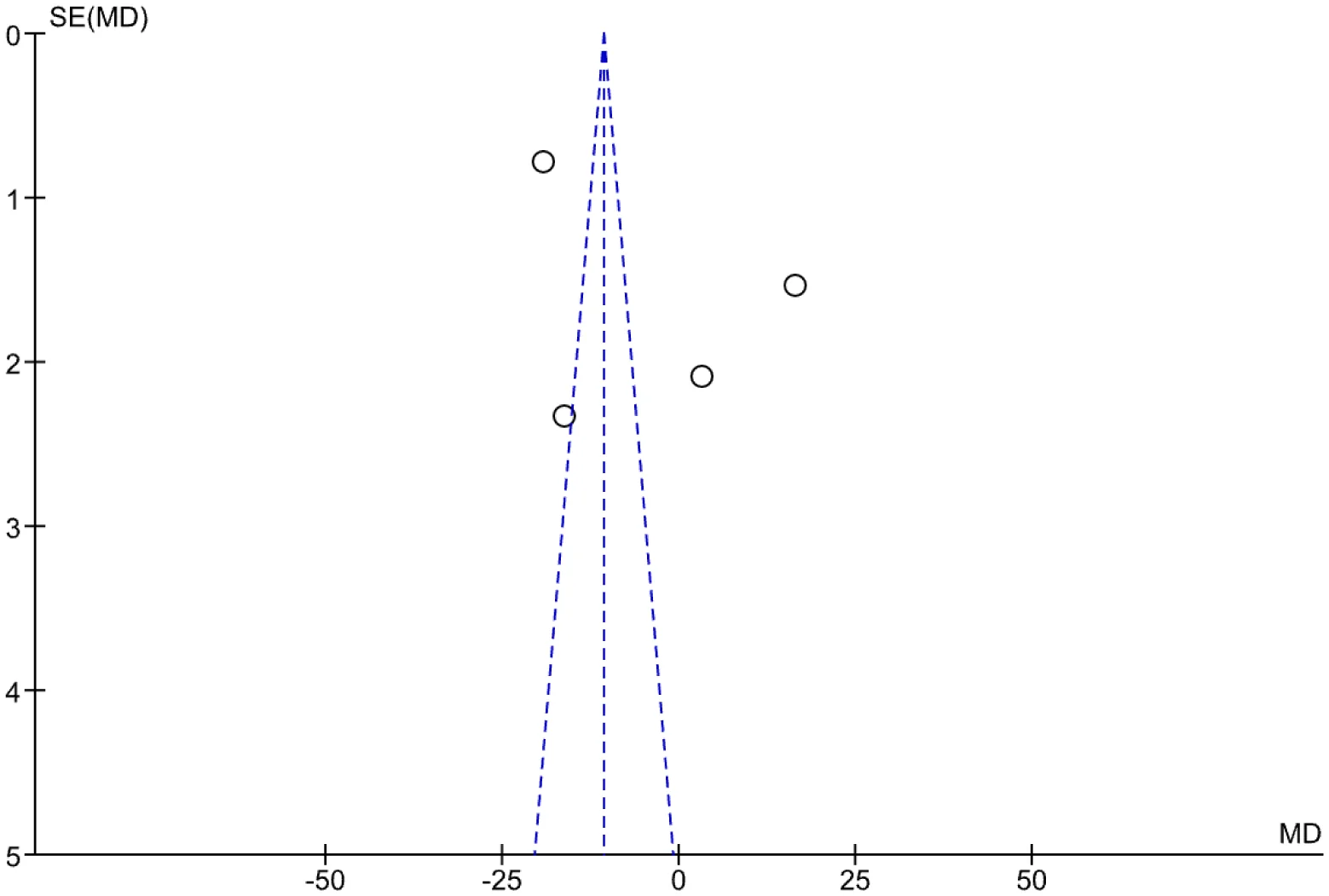
Funnel plot used to evaluate the existence of publication bias. The funnel plot showed a wide variability in the data.

Figures 3 and 4 display the funnel and forest plots, respectively, and show a wide variability in the data; despite this, the results favor the experimental group. In fact, the greatest weight (66.01%) comes from the study by Dai et al., [41], which has a low standard deviation for the experimental group (3.95) and minimal variability. The data is significant as it favors the experimental group. Moreover, we used funnel plots to assess any potential publication bias, which highlights the heterogeneity of the other studies. The treatments are associated with greater alleviation of negative symptoms; despite this, this meta-analysis has some limitations. First, this meta-analysis may have been underpowered due to the heterogeneity of the studies and the small sample of participants. Second, although most of the RCTs included a sham control in the study design, all of the included studies were relatively small trials, with small samples and heterogeneous technical procedures.

## 4. Discussion

This is the first review and meta-analysis to evaluate the effect of different types of NIBS techniques on suicidal ideation.

Three studies regarding TMS [38],[41],[42] showed a statistically significant decrease in suicidal ideation. Two studies [37],[39] described a clinical improvement in suicidal ideation, though with no significance. Only Yesavage et al., [40] observed non-significant findings for measures of suicidality at both the end of acute treatment and the follow-up phases; the absence of significant results in this study may be due to the relatively short stimulation protocol, as well as the older age of the participants, which are factors that could limit neuroplastic effects and clinical responses. All these studies explored suicidality in the context of a depressive disorder; this fact limits the conclusions of the data only to suicide depression symptoms and not to suicidal ideation in general. All studies applied high-frequency stimulation, including TBS studies, and no studies examined the effect of low frequency stimulation, thus making it difficult to speculate on the relationship between TMS frequency and suicidality. Although the result of our review seems to show that high-frequency TMS decreased suicidality, we may have observed a protective effect and an exerted emotional or cognitive control, as previously reported [47].

Regarding dTMS treatment, Berlim et al., [43] observed a significant decrease in the HAM-D scores, thus showing a possible reduction of suicidal ideation in the context of depressive symptomatology. This study agrees with the literature about rTMS, which show how this procedure significantly reduces suicidal ideation in depressed patients [48]. Kaster et al., [44] drew attention to a reduction in suicide ideation in depressed patients after dTMS, thus confirming the effectiveness of deep TMS in treating depression but not suicidality. Other studies explored the efficacy of dTMS in suicide ideation, but with a lower statistical power than RCT. For example, Rapinesi et al., [49]showed a reduction in suicidal ideation at the end of treatment in a single patient with depression and alcohol use disorder, thus suggesting that dTMS may be effective in treating both depressive symptoms and addiction. However, due to the lack of data, it is not possible to evaluate if dTMS can be a useful strategy in treating suicide ideation, in different types of psychiatric disorders. Further studies, especially RCT, are needed to compare deep TMS and other NIBS techniques.

Finally, the study that evaluated tDCS showed a protective effect on suicidality compared with the placebo, thus demonstrating how tDCS decreased suicidal ideation significantly. Nevertheless, knowledge of how such a method affects the circuits involved in suicidality requires further research. Indeed, the current evidence on tDCS is insufficient to conclusively determine its efficacy due to the limited number of randomized controlled trials and small sample sizes.

Our results seem to provide an indication of the use of NIBS on suicidality, although further studies are needed to better explore the use of the technique in this area. The observed NIBS action on suicidality could be linked to the plasticity-inducing effect, since plasticity can promote the neuro-regeneration that is impaired in depression [50].

Nevertheless, the statistically significant differences between the control group and the active group observed in studies reporting suicide ideation reduction might also indicate a possible placebo effect of NIBS, especially rTMS. Indeed, this hypothesis agrees with studies in the literature which showed that TMS had a noticeable “placebo effect” in the treatment of various disorders [48],[51].

Among the included studies, sham procedures differed in their complexity and efficacy in maintaining blinding. George et al., [37] and Desmyter et al., [38] employed active sham rTMS using coil placements or inserts that replicated the auditory click and scalp sensations of real stimulation but prevented actual cortical activation. This approach enhances blinding by mimicking active treatment more closely. Dai et al., [41] and Pan et al. [42] also used active sham, and either rotated the coil slightly away from the head or employed angled positioning to preserve sensory cues without delivering significant stimulation. Baeken et al., [39] and Kaster et al., [44] utilized passive sham dTMS by either using a sham coil or positioning the coil with minimal actual stimulation, which reduces participant discomfort but may risk unblinding. Yesavage et al., [40] used a passive sham for rTMS and likely involved diminished stimulation without matching sensations, thus potentially permitting participants to distinguish between conditions. Berlim et al., [43] used passive sham by positioning the coil away from the skull without replicating sensations, thus potentially weakening blinding. Finally, in the Brunoni et al., [45] tDCS trial, active sham tDCS was used; a brief current ramp-up mimicked the tingling sensations of real stimulation before turning off, which is a method shown to better preserve subject blinding. This variability in sham approaches can impact blinding effectiveness and may influence the magnitude of the placebo response. By preserving sensory cues, active sham tends to enhance blinding but is more complex to implement; while simpler, passive sham risks unblinding and potentially inflating placebo effects. The variability in sham methods could partially account for inconsistent findings and complicates the interpretation of treatment effects. Therefore, future studies should standardize and report sham procedures in detail and ideally include blinding checks to evaluate the credibility of the placebo condition.

This review has important limitations. The first limitation regards the fact that only 9 studies, which often had small sample sizes, were included in this review, and suicidality was the primary outcome considered, though wasn't the focus of all articles. One issue was the heterogeneity among the studies, which was moderate to high in most analyses. The substantial heterogeneity observed across the included studies may be attributed to several methodological and clinical factors. First, the neuromodulation protocols widely varied in terms of the stimulation parameters, including frequency (e.g., 10 Hz, 18 Hz), intensity (e.g., 110% vs. 120% of RMT), duration (e.g., daily vs. accelerated protocols), and number of sessions. Second, differences in the patient populations likely contributed to the variability: for instance, some studies included younger adults, while others focused on elderly participants, and the diagnostic criteria also varied (e.g., unipolar depression, treatment-resistant depression). Third, the outcome measures for suicidality were inconsistent, and ranged from item-based assessments within depression scales (e.g., MADRS) to validated suicide-specific instruments (e.g., SSI, BSI, SIOOSS). Another limitation regards the fact that only studies involving patients with depressive disorders were selected; other analysis extended to all psychiatric disorders should be considered, as suicide is a transversal issue in Psychiatry. In addition, only one study on tDCS met the inclusion criteria, which limits the generalizability of findings related to this technique. Finally, the exclusion of databases such as EMBASE or PsycINFO might have limited the comprehensiveness of the literature search.

Future research should aim to standardize neuromodulation protocols, including stimulation parameters and targeted brain regions, to improve the comparability across studies. Additionally, more investigations are needed on populations beyond MDD, such as individuals with bipolar disorder, schizophrenia, or substance use disorders, to address the transdiagnostic nature of suicidality. Larger, multicenter randomized controlled trials with longer follow-up periods are necessary to evaluate the durability of the treatment effects. Finally, the integration of neurobiological markers could help identify patients who are most likely to benefit from NIBS interventions aimed at reducing suicidal ideation and behavior.

## 5. Conclusions

The WHO's recent report [12] showed high rates of suicide worldwide between 2009 and 2019. In addition, this report highlighted that the recent Covid-19 pandemic has been described as an environmental factor associated with increased suicide risk. Indeed, at the beginning of the pandemic in all parts of the world, suicides related to the social phenomenon of the SARS-CoV-2 virus were recorded, underlining how the treatment together with the prevention of suicidality could be considered a public health priority.

In this context, NIBS techniques might represent promising tools to reduce suicidal ideation. However, the current evidence is still limited. Future studies should aim to standardize the stimulation protocols, include non-depressive and transdiagnostic populations, adopt longer follow-up periods, and incorporate biological markers to predict treatment response. These efforts will be essential to determine the clinical utility of NIBS in addressing this pressing global mental health challenge.

## Use of AI tools declaration

The authors declare they have not used Artificial Intelligence (AI) tools in the creation of this article.


