# Supplementary material for: Non-invasive brain stimulation for suicidal ideation: a systematic review and metanalysis of the current literature
Source: AIMS Neurosci. 2025 Jul 25;12(3):332–50. doi: 10.3934/Neuroscience.2025018 (PMC12521931; doi:10.3934/Neuroscience.2025018)
Supplement: Supplementary file 1 [file neurosci-12-03-018-s001.pdf]

AIMS Neuroscience

AIMS Neuroscience, 12(3): 332–350.
DOI: 10.3934/Neuroscience.2025018
Received: 12 June 2025
Revised: 08 July 2025
Accepted: 16 July 2025
Published: 25 July 2025

https://www.aimspress.com/journal/neuroscience

Review

Non-invasive brain stimulation for suicidal ideation: a systematic review and metanalysis of the current literature

Fiammetta Iannuzzo, Fabrizio Turiaco, Vincenzo Messina, Alessandro Magazzù Minutoli, Maria Catena Silvestri, Maria Rosaria Anna Muscatello and Antonio Bruno*

Department of Biomedical and Dental Sciences and Morphofunctional Imaging, University of Messina, Via Consolare Valeria 1, Contesse, Messina 98125, Italy

* **Correspondence:** Email: antonio.bruno@unime.it.

Supplementary

Full Search Strategy for Each Database:

- *PubMed*

("Transcranial Magnetic Stimulation" [Mesh] OR "repetitive Transcranial Magnetic Stimulation" OR rTMS OR TMS OR "deep TMS" OR "transcranial direct current stimulation" OR tDCS OR "non-invasive brain stimulation" OR NIBS)

AND

("Suicide" [Mesh] OR suicide OR suicidality OR "suicidal ideation" OR "suicidal thoughts" OR "suicidal behavior").

- *Scopus*

TITLE-ABS-KEY ("transcranial magnetic stimulation" OR TMS OR rTMS OR "deep TMS" OR "transcranial direct current stimulation" OR tDCS OR "non-invasive brain stimulation" OR NIBS)

AND

("suicide" OR "suicidality" OR "suicidal ideation" OR "suicidal thoughts" OR "suicidal behavior").

- *Web of Science*

TS = ("transcranial magnetic stimulation" OR TMS OR rTMS OR "deep TMS" OR "transcranial direct current stimulation" OR tDCS OR "non-invasive brain stimulation" OR NIBS)

AND

TS = (“suicide” OR “suicidality” OR “suicidal ideation” OR “suicidal thoughts” OR “suicidal behavior”).

AIMS Press

© 2025 the Author(s), licensee AIMS Press. This is an open access article distributed under the terms of the Creative Commons Attribution License (https://creativecommons.org/licenses/by/4.0)
